# Redox signalling and ageing: insights from *Drosophila*

**DOI:** 10.1042/BST20190052

**Published:** 2020-03-20

**Authors:** Claudia Lennicke, Helena M. Cochemé

**Affiliations:** 1 MRC London Institute of Medical Sciences, Du Cane Road, London W12 0NN, U.K.; 2 Institute of Clinical Sciences, Imperial College London, Hammersmith Hospital Campus, Du Cane Road, London W12 0NN, U.K.

**Keywords:** ageing, *Drosophila*, redox signalling

## Abstract

Ageing and age-related diseases are major challenges for the social, economic and healthcare systems of our society. Amongst many theories, reactive oxygen species (ROS) have been implicated as a driver of the ageing process. As by-products of aerobic metabolism, ROS are able to randomly oxidise macromolecules, causing intracellular damage that accumulates over time and ultimately leads to dysfunction and cell death. However, the genetic overexpression of enzymes involved in the detoxification of ROS or treatment with antioxidants did not generally extend lifespan, prompting a re-evaluation of the causal role for ROS in ageing. More recently, ROS have emerged as key players in normal cellular signalling by oxidising redox-sensitive cysteine residues within proteins. Therefore, while high levels of ROS may be harmful and induce oxidative stress, low levels of ROS may actually be beneficial as mediators of redox signalling. In this context, enhancing ROS production in model organisms can extend lifespan, with biological effects dependent on the site, levels, and specific species of ROS. In this review, we examine the role of ROS in ageing, with a particular focus on the importance of the fruit fly *Drosophila* as a powerful model system to study redox processes *in vivo*.

## Introduction

According to the World Health Organization (WHO), global life expectancy has increased steadily over recent decades reaching 72 years in 2016 [1]. A girl born in 2012 can expect to live on average until 73, a boy until 68, which is 6 years longer than the average global life expectancy for a child born in 1990 [2]. This impressive rise is due to many factors, such as improvements in living standards, sanitation, nutrition and medical advances. However, this achievement of humanity is linked to a down-side — an ageing population. Increasing age is a risk factor for many diseases, such as type-2 diabetes, cardiovascular disorders, cognitive degeneration, immune dysfunction, as well as different types of cancer [3,4]. Ageing also increases the risk of morbidity and mortality from infectious diseases, and the susceptibility to injury and trauma due to the impairment of balance and mental state [4]. Consequently, age-related diseases have become the greatest health threat of the 21st century. Ageing is therefore widely regarded as the most critical socio-economic challenge for the next decades [4]. Understanding the biological mechanisms underlying the ageing process itself and the development of age-related diseases is key to combatting this crisis.

The ageing of multicellular organisms can be described as the loss of cellular function over time, resulting in the failure to maintain systemic homeostasis, and the progressive decline of physiological integrity [5]. A series of ‘hallmarks’ have been proposed as contributors to the ageing process: genomic instability, telomere attrition, epigenetic alterations, loss of proteostasis, deregulated nutrient sensing, mitochondrial dysfunction, cellular senescence, stem cell exhaustion, and altered intracellular communication [5]. Although numerous hypotheses have been proposed, the nature and causes of ageing are still poorly understood. The long-established ‘free radical theory of ageing’ postulates that free oxygen radicals, generated during normal cellular metabolism, play a key role in the ageing process by randomly oxidising and thereby damaging biomacromolecules, such as lipids, DNA, and proteins [6]. Previous studies have suggested that the over-production of reactive oxygen species (ROS) may have complex roles in promoting the development of age-related diseases [7]. In addition, the cellular elevation of ROS has been associated with the onset and progression of ageing [8]. Meanwhile, it is widely accepted that ROS not only cause oxidative damage, but are also involved in normal cellular function as signalling molecules, through the oxidation of functional thiols on target proteins [9,10]. According to this hypothesis, the accumulation of ROS over time would lead to a pro-oxidising shift of cellular thiols with age, resulting in the disruption of normal redox signalling processes. The state of oxidative stress can be triggered by distinct mechanisms: enhanced ROS production, insufficient antioxidant capacity, and/or increased uptake of environmental pro-oxidants [11]. To explore the existence of a relationship between the ageing process and a shift in cellular redox homeostasis, the application of advanced redox techniques to *in vivo* model systems is essential. The aim of this article is to review current knowledge on the connection between ROS, redox signalling and ageing, focussing on the advances achieved using the fruit fly *Drosophila melanogaster*.

## *Drosophila*, a powerful model to study ageing

*Drosophila* is an outstanding model to study the complexity of ageing. *Drosophila* has a comparatively short tractable lifespan of ∼3 months at an ambient temperature of 25°C (compared with ∼3 years for mice), and exhibits many recognisable features of ageing (e.g. decreased physical and metabolic activity, increased sensitivity to stress, declined reproductive capacity, disrupted sleep) [12]. Critically, key metabolic pathways involved in the ageing process, such as insulin/IGF-1-like signalling and target-of-rapamycin (TOR) signalling, are strongly evolutionarily conserved between *Drosophila* and mammals [12]. Moreover, a large number of powerful genetic tools are available, including CRISPR-Cas9 and the UAS/GAL4 expression system, enabling the efficient manipulation, identification and characterisation of single-gene mutations that extend lifespan. These genetic techniques allow not only the temporal control of genetic alterations, but also open the possibility to study the ageing process in a tissue-specific manner. Importantly, *Drosophila* is an excellent model to investigate the connection between cellular redox state and ageing, as the tissues of adult flies are composed of post-mitotic cells, therefore senescent changes are not diluted by successive cell division [13]. In addition, as a poikilotherm, metabolic rate and life expectancy in *Drosophila* can be easily altered by varying the ambient temperature — for instance, flies are significantly longer lived when raised at 18°C compared with 25°C [14,15]. In this context, lifespan extension at cold temperatures is not only observed in poikilotherms but also in homeotherms, including mammals [16,17].

## The connection between oxidative stress, redox signalling and ageing

The so-called ‘free radical theory of ageing’ was proposed in the 1950s, and postulates that ROS generated during normal cellular metabolism play a key role in the ageing process [6]. This theory was subsequently modified to the ‘oxidative stress theory of ageing’ because peroxides, such as hydrogen peroxide (H_2_O_2_), that are technically not free oxygen radicals, can also cause oxidative damage [18]. Both theories presuppose that the rate of ROS production outweighs the rate of ROS elimination by endogenous antioxidant defence systems. Excess ROS can randomly damage biomacromolecules (i.e. lipids, DNA and proteins), that accumulate over time within cells leading to the disruption of normal cellular functions. For instance, upon reaction with ROS, carbohydrates can generate highly reactive carbonyl compounds, such as glyoxal and methylglyoxal, which can form stable adducts with lysine residues on proteins [19]. Furthermore, lipid peroxidation products can react with proteins, forming *N*-malondialdehyde-lysine. ROS can also attack DNA producing over 20 different base lesions, with 8-hydroxy-2′-deoxyguanosine being the most extensively studied and used as a biomarker for oxidative DNA damage [20]. Strikingly, an increase in the steady-state level of oxidatively damaged molecules over time, including a variety of specific protein and carbohydrate adducts, has been reported in various species, including *Drosophila* [19,21,22]. However, whether this accumulation of oxidative damage with age is causal or simply correlational remains unclear.

**Figure 1.**
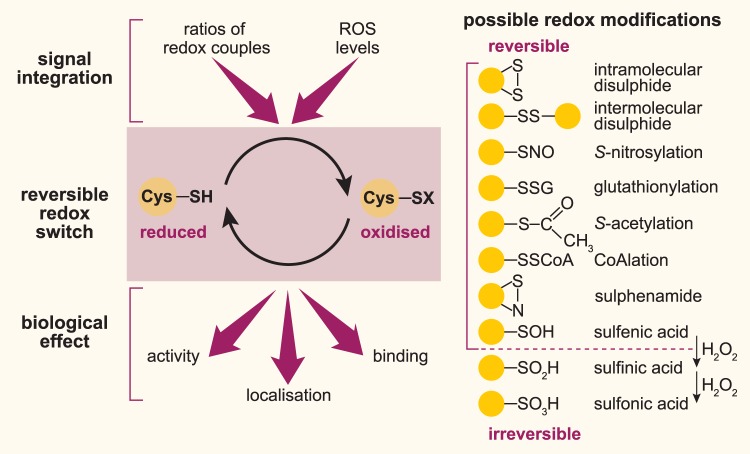
Scheme of redox signalling and possible cysteine-based redox modifications. Redox signalling integrates cues from the redox ratios of cellular redox cofactors (e.g. glutathione, NADPH) and the levels of ROS (e.g. H_2_O_2_). The reversible redox switch operates through a post-translational modification of thiol groups on cysteine residues, which induces a biological response in the target protein (e.g. change in enzyme activity, binding interactions, sub-cellular localisation). Redox-sensitive cysteine residues are present in the thiolate form under physiological conditions and are therefore prone to redox modifications by H_2_O_2_. A wide range of reversible cysteine-based redox modifications are possible, representing a targeted signalling event, which is distinct from irreversible oxidative damage.

Meanwhile, it is widely accepted that besides playing a role in molecular damage, ROS fulfil second messenger-like functions. H_2_O_2_ is produced enzymatically for instance by superoxide dismutase (SOD) or the NADPH oxidase (NOX) system, and exhibits low reactivity overall, but a relatively high selectivity towards proteins containing thiol groups [10]. H_2_O_2_ mediates the post-translational oxidative modification of specific cysteine residues, which can alter the function, activity or localisation of the target protein (Figure 1). Here, the deprotonated cysteine residue (thiolate) functions as a redox-sensitive switch, and H_2_O_2_ acts as the signalling agent, although how H_2_O_2_ selectively and efficiently oxidises specific thiol residues is still elusive. Increasing evidence supports a role for thiol peroxiredoxins (Prxs), which account for ∼1% of cellular protein content, as mediators of protein thiol oxidation via so-called intracellular redox signalling relays, rather than H_2_O_2_ acting as a direct messenger [23]. In *Drosophila*, the Prx family consists of seven members, which like their mammalian orthologues reside in different sub-cellular compartments [18]. Recently, the cytosolic Prx Jafrac1 was shown in *Drosophila* S2R+ cells to form a transient mixed disulfide with Mekk1, the fly homologue of mammalian MEKK4, in response to H_2_O_2_ [24]. MEKK4 is the main MAP3K responsible for initiating H_2_O_2_-induced activation of p38. Redox-dependent interaction with Jafrac1 led to oxidation and activation of Mekk1, which subsequently phosphorylated p38 [24]. To which extent this redox relay occurs in *Drosophila* under physiological and/or stressed conditions needs to be investigated.

The range of reversible thiol modifications is diverse and includes sulfenylation, intra- or intermolecular disulfides, glutathionylation, nitrosylation, as well as acetylation and CoAlation (Figure 1) [10,25,26]. In this context, the formation of glutathionylated proteins by the reaction of glutathione (GSH) with sulfenic acid is regarded as a mechanism to protect proteins against over-oxidation [10]. All these modifications are reversible and can easily be reduced by the GSH and thioredoxin (Trx) based antioxidant systems. However, in the presence of high H_2_O_2_ levels, sulfenic acid is prone to irreversible over-oxidation, resulting in sulfinic (R-SO_2_H) and sulfonic (R-SO_3_H) acid modifications (Figure 1). GSH plays a role not only by directly protecting cysteine residues of proteins from irreversible over-oxidation, but is also involved in maintaining cellular redox state as part of the GSH/GSSG redox couple. In mammals, the regeneration of the thiol reductant GSH relies on the enzyme glutathione reductase (GR). In contrast, *Drosophila* lacks GR and is therefore dependent on the thioredoxin reductase (TrxR) system to recycle GSH from GSSG [27].

The oxidation of GSH, cysteine, and methionine are some of the earliest cellular responses to an increase in ROS production, which as a consequence lead to alterations in cellular redox potential [28]. Several studies have been undertaken in flies to investigate if the GSH/GSSG ratio changes with age. A direct comparison of two different *Drosophila* strains (*Oregon R* and *yw*) that vary in longevity indicated that the amount of GSSG increased whereas the GSH/GSSG ratios declined as a function of age in both strains [13]. Interestingly, the shorter-lived strain exhibited a more rapid decline in GSH/GSSG ratio compared with the longer-lived strain. However, the experimental enhancement of oxidative stress by exposing the flies to hyperoxia (100% O_2_) neither reproduced nor accelerated the pattern of alterations in the glutathione redox state observed during ageing under normoxic conditions [13]. Another study similarly reported that the ratio of GSH/GSSG and methionine decreased during ageing in *Drosophila*, whereas the protein mixed disulfide content increased [29]. Temperature is known to have a strong impact on the metabolic rate and lifespan of flies. A direct comparison of flies kept under temperatures ranging from 18°C to 30°C indicated that the amount of mixed disulfides and GSSG increased at higher temperatures [29]. However, the GSH/GSSG ratios were measured in lysates obtained from whole flies, meaning the obtained GSH/GSSG ratios are averaged over tissues, cell types and sub-cellular compartments. Furthermore, the GSH/GSSG redox couple alone cannot be considered as representative for all redox processes that occur in a cell at a given time [30]. For example, an increase in mitochondrial GSSG levels might have different causes and also consequences than an increase in mitochondrial H_2_O_2_ levels [30].

If ROS/H_2_O_2_ and altered redox status are indeed a causal factor of ageing, the activity of antioxidants should negatively correlate with age. However, studies in *Drosophila* and several other species have indicated that there is no generalised age-related trend in activities of various antioxidant enzymes [18]. Some enzymes showed a decline during ageing, some an up-regulation, whereas others showed no difference in their activity levels in aged compared with young flies [18]. Nevertheless, the administration of dietary antioxidants or the genetic overexpression of antioxidant enzymes involved in the detoxification of ROS should theoretically combat ageing and/or age-related diseases and should therefore extend lifespan.

Transgenic flies carrying an additional copy of either CuZn-SOD (SOD1) or MnSOD (SOD2), responsible for degrading superoxide (O_2_^•−^) in the cytosol and mitochondria respectively, or catalase, which degrades H_2_O_2_, exhibited no benefits regarding lifespan [31,32]. Flies co-expressing SOD1 and catalase were initially reported to be long-lived [33], however a later study found these effects to be associated with genetic background, and that overexpression of SOD1, SOD2, catalase and TrxR, either alone or in combination, failed to increase fly survival [34]. Furthermore, expression of ectopic mitochondria-targeted catalase generally conferred resistance against oxidative stress, but did not affect or even shortened lifespan in *Drosophila* [35–37]. In contrast, overexpression of SOD1 specifically in adult fly motoneurons resulted in an extension of lifespan by up to ∼40% and rescued the survival of a short-lived SOD1-null mutant [38]. In addition, several studies have shown that dietary supplementation with antioxidants (e.g. vitamin E) failed to extend fly lifespan [39]. These mixed results therefore led to a questioning of the causality between increased ROS levels and ageing.

Overexpression of the cytosolic Prx Jafrac1 specifically in neuronal tissue conferred resistance to oxidative stress induced by paraquat, as well as lifespan extension under untreated conditions [40]. The authors found reduced ROS levels in the brains of Jafrac1 overexpressing flies and restored ATP production upon paraquat treatment, leading to the conclusion that Jafrac1 may act as a protector of neuronal mitochondria under oxidative stress conditions by attenuating JNK/FOXO signalling [40]. Strikingly, the selective overexpression of dPrx5 in neuronal tissues did not exhibit an effect on fly survival [41], potentially indicating a specific role for neuronal Jafrac1 in modulating lifespan. However, the ubiquitous overexpression of dPrx5 extended survival of flies by ∼30% [41]. Interestingly, dPrx5 has multiple sub-cellular localisations, including the cytosol, nucleus and mitochondria, whereas dPrx3 is found solely in mitochondria. The combined (but not individual) knock-down of dPrx3 and dPrx5 led to a strong decrease in the GSH/GSSG ratio and in reduced protein thiol levels, resulting in pro-apoptotic changes and dramatically shortened lifespan [42]. Furthermore, overexpression of dPrx5 targeted to mitochondria, but not to the cytosol or nucleus, partially rescued the shortened survival of dPrx3 and dPrx5 double knock-down mutants under both stressed and control conditions [43]. The authors implicate chronic activation of immune responses, highlighting an important role for mitochondrial redox regulation in this process [43].

**Figure 2.**
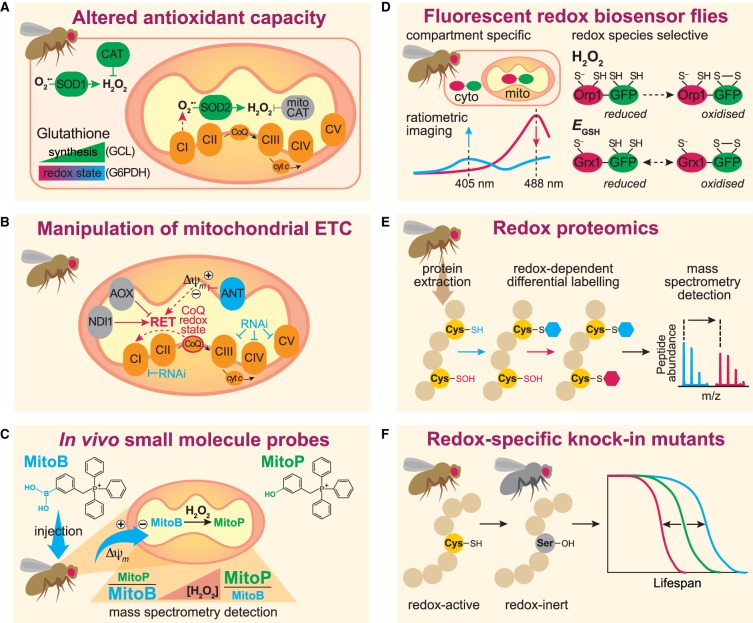
Evaluating the role of ROS in the ageing process using *Drosophila* as a model organism. (**A**) Different genetic strategies to manipulate antioxidant capacity and redox state in the context of lifespan. These include the overexpression of endogenous (e.g. SOD1, SOD2, CAT) and ectopic (e.g. mito-CAT) antioxidant enzymes. Altered redox state can be achieved by manipulating the redox cofactor glutathione, for instance by overexpressing GCL (glutamate-cysteine-ligase) to enhance synthesis, or indirectly by overexpressing G6PDH (glucose-6-phosphate dehydrogenase), which leads to increased NADPH, an important reducing equivalent for the recycling of antioxidant systems. (**B**) Manipulation of the mitochondrial electron transport chain (ETC). ROS production through reverse electron transport (RET) can extend lifespan in flies, and requires a highly reduced coenzyme Q (CoQ) pool. Elevated mitochondrial membrane potential (Δψ_m_) also drives RET, which can be dissipated for instance by overexpression of the adenine nucleotide translocase (ANT). Ectopic expression of an internal NADH dehydrogenase (e.g. NDI1) or an alternative oxidase (AOX) can enhance or abolish RET, respectively. (**C**) Use of a small molecule probe to measure mitochondrial H_2_O_2_ levels *in vivo*. MitoB is a lipophilic cation, which following injection into the fly accumulates in the matrix of mitochondria driven by the membrane potential. The boronic acid moiety of MitoB reacts stoichiometrically with H_2_O_2_ to form a phenol product, MitoP. The MitoP/MitoB ratio can be determined from extracted fly samples and accurately measured by mass spectrometry against deuterated internal standards to give mitochondrial H_2_O_2_ levels. (**D**) Genetically encoded fluorescent redox-sensitive biosensors allow compartment-specific (cytoplasmic or mitochondrial) or redox species-selective (H_2_O_2_ or glutathione redox potential, *E_GSH_*) visualisation and estimation of ROS/redox state with information on tissue distribution. (**E**) To identify redox-sensitive cysteine residues, state-of-the-art redox proteomic approaches can be applied. Differential labelling of free (reduced) cysteines is initially performed using a first thiol-specific tag. All reversibly modified (oxidised) cysteines are then reduced *in vitro* with a reducing agent (e.g. DTT or TCEP), and labelled with a second tag (e.g. containing a heavy isotope, which causes a mass shift). Labelled proteins are digested, enriched for the tagged peptides, and finally subjected to analysis by mass spectrometry. (**F**) The role of redox regulation for a specific cysteine residue on physiology, metabolism and ageing can be dissected by generating targeted redox knock-in mutant flies, where the redox-active cysteine residue has been replaced by a redox-inert residue (e.g. serine).

Besides antioxidant enzymes directly involved in the detoxification of ROS, another strategy to manipulate cellular redox state *in vivo* is to target enzymes that provide reducing equivalents for the recycling of antioxidant systems and redox couples. For instance, overexpression of glutamate-cysteine-ligase (GCL), which catalyses the rate-limiting step in the synthesis of GSH, increased lifespan in flies [44,45]. Similarly, overexpression of glucose-6-phosphate dehydrogenase (G6PDH), an enzyme belonging to the pentose phosphate pathway (PPP) involved in the generation of NADPH, robustly extended fly lifespan [46]. Overall, manipulating the expression of enzymes that are involved in maintaining cellular redox homeostasis by providing reducing equivalents appears to significantly prolong fly lifespan (Figure 2A). Therefore, redox signalling processes, rather than antioxidant enzymes, might play a role in modulating the ageing process [18,47].

## The role of mitochondria

The site of ROS generation plays an important role in determining its cellular effects. ROS production occurs as a by-product cellular respiration, and as a consequence O_2_^•−^ is predominantly generated by mitochondria [48,49]. Signalling proteins that are involved in mediating apoptotic cell death, e.g. caspase-3 and caspase-9, are redox-sensitive and can be found in the intermembrane space of mitochondria [50–53]. Accumulation of damage mediated by mitochondrial ROS has been proposed as a major driver of ageing [54]. For instance, a decline in mitochondrial function has been associated with normal ageing and is furthermore correlated with the development of age-related diseases [55–57]. The majority of reports have found that ageing is generally accompanied by a decreased activity of mitochondrial enzymes (e.g. citrate synthase), and an increase in ROS production [58]. In fact, the age-related increase in mitochondrial ROS has been proposed as a cause of the ageing process [59], whereas mitochondrial dysfunction is viewed as a hallmark of ageing [5].

Mitochondrial DNA (mtDNA) mutations increase with age, as shown in both animal models and humans [58]. To address the question if mtDNA mutations have an impact on ageing, a recent study engineered flies expressing a mutant version of the catalytic subunit of the mtDNA polymerase, thus introducing mtDNA mutations. Interestingly, in contrast with a previous study showing that even low levels of mtDNA mutations caused developmental delays in flies [60], mtDNA mutations did not broadly affect the lifespan or healthspan of adult flies [61].

Several lines of evidence implicate mitochondrial ROS in longevity. A number of studies have found an inverse correlation between the rate of mitochondrial ROS production and lifespan in several different species [53]. However, the current opinion is that the level of ROS, the specific type of ROS, and the site of ROS generation determine the impact on physiological function [9,62–64]. In this context, low levels of ROS may stimulate mitohormesis, whereas higher levels might lead to oxidative damage [65].

Multiple studies have independently shown that increased ROS levels in animal models, including *Drosophila*, extend lifespan [30,66,67]. Assays with isolated *Drosophila* mitochondria indicate that complex I and glycerol-3-phosphate dehydrogenase are the predominant sites of O_2_^•−^ generation *in vitro* [68,69]. To resolve site-specific production of ROS and manipulate levels of ROS *in vivo*, alternative respiratory enzymes that are absent in mammals or flies have been used (Figure 2B) [65–67,70,71]. Alternative NADH dehydrogenases, which are able to oxidise NADH from the mitochondrial matrix and directly reduce coenzyme Q (CoQ) without pumping protons across the inner mitochondrial membrane, can effectively by-pass complex I of the conventional mitochondrial electron transport chain (ETC). In this context, allotopic expression of NDI1, a rotenone-insensitive alternative NADH dehydrogenase found in plants and fungi, caused a significant elevation of mitochondrial ROS and extended fly lifespan [67]. Interestingly, co-expression of mitochondria-targeted catalase abolished the lifespan extension conferred by NDI1, confirming that the increased longevity was a result of ROS production via reverse electron transport (RET), driven under conditions of a highly reduced CoQ pool and elevated mitochondrial membrane potential [67]. Similarly, expression of NDX, an alternative NADH dehydrogenase from the tunicate *Ciona intestinalis*, significantly extended *Drosophila* lifespan both on a control diet [71] and on a range of protein-to-carbohydrate ratios [70]. Additionally, expression of an alternative oxidase (AOX) enzyme from *C. intestinalis*, able to reduce O_2_ to H_2_O with electrons received from CoQ thus by-passing complexes III and IV of the ETC, also prevented RET by oxidising the CoQ pool and abolished the lifespan extension of NDI1 flies [67]. Consistent with other *in vivo* studies [30,72], the authors show that mitochondrial ROS production increases with age in *Drosophila* [67]. Non-detoxified ROS can be detrimental to *Drosophila* survival, while ROS production via RET, specifically from the reduced CoQ pool, acts as a signal to maintain mitochondrial function and extend lifespan [67].

RET is heavily dependent on a high mitochondrial membrane potential as a driving factor [73]. Flies overexpressing mitochondrial adenine nucleotide translocase (ANT) are characterised by increased proton conductance over the mitochondrial inner membrane, therefore these flies were used as a model to study the impact of mitochondrial membrane potential on ROS production. ANT overexpressing flies were shorter-lived compared with control flies and also showed lower ROS production [73], consistent with the importance of RET to promote longevity, and that decreasing mitochondrial ROS production does not necessarily extend lifespan.

Another strategy to manipulate mitochondria *in vivo* involves the RNAi of ETC subunits. Knock-down of genes encoding components of mitochondrial respiratory complexes I, III, IV and V led to increased lifespan in *Drosophila* [74]. Although these long-lived flies did not show reduced total ATP levels, suggesting the effects on lifespan are uncoupled from energy production, other potentially relevant parameters such as mitochondrial respiration and ROS production were not reported. Therefore, the exact mechanism underlying the lifespan extension of these ETC RNAi flies, and whether redox signalling is involved, remains to be fully elucidated.

## Assessment of ROS production and redox state *in vivo*

Much data obtained on ROS production in *Drosophila* was originally conducted on isolated mitochondria [68,69] or whole fly lysates, averaging the impact of tissue-specific and cellular compartment-specific effects of altered redox status. In addition, most popular probes targeting ROS are prone to artefacts. For instance, 2′,7′-dichlorofluorescein diacetate (DCFDA) reacts non-specifically with many forms of ROS [75]. Moreover, MitoSOX, a fluorescent probe frequently used for the detection of mitochondrial O_2_^•−^, was found to cause mitochondrial dysfunction with the potential to alter O_2_^•−^ production [76].

Recently more defined approaches have been developed to measure ROS *in vivo* under physiological conditions. These include the mitochondria-targeted ratiometric probe MitoB [72], containing a boronic acid moiety selective for H_2_O_2_, which is analysed by mass spectrometry enabling sensitive quantification (Figure 2C). MitoB was applied to *Drosophila* to show that mitochondrial H_2_O_2_ levels increased *in vivo* with age [72]. However, this age-related increase in mitochondrial H_2_O_2_ was not co-ordinately altered by interventions that regulate lifespan, such as dietary restriction or physical activity [72].

In parallel, the development of genetically encoded probes based on redox-sensitive GFPs (roGFPs) allowed imaging of ROS levels and the redox state with tissue-specific and sub-cellular resolution [77]. These redox-sensitive GFP probes contain an engineered disulfide switch, which is associated with a ratiometric shift in fluorescence (Figure 2D). The usage of transgenic *Drosophila* lines expressing different redox probes allowed the measurement of *E*_GSH_ and H_2_O_2_ levels in both the cytosolic and mitochondrial compartments. The authors reported that transgenic larvae exhibited natural differences between different cell and tissue types. Interestingly, both age-dependent and age-independent oxidation processes occurred in different tissues of the adult fly [30]. A key finding was that ageing-dependent pro-oxidant changes existed but were highly restricted to specific regions, rather than affecting the whole organism. For instance, midgut enterocytes are major sites of cytosolic H_2_O_2_ accumulation during ageing [30]. In addition, results obtained with the H_2_O_2_-specific Orp1-roGFP and GSH-specific Grx1-roGFP probes revealed that changes in the H_2_O_2_ status and GSH redox can be uncoupled [30]. Thus, the sole measurement of only one or the other might lead to misinterpretations. Strikingly, lifespan extension was accompanied by increased formation of oxidants, rather than by a decrease [30]. This imaging method allows the investigation of the *in vivo* influence of pharmaceuticals with antioxidant potential. *N*-acetyl-cysteine (NAC) was previously shown to extend the lifespan of flies [78]. However, feeding transgenic roGFP-expressing flies with NAC had no influence on H_2_O_2_ levels or GSH redox state, thus questioning the *in vivo* antioxidant activity of NAC [30].

Further technological advances allow the proteomic assessment of thiol redox state, or the ‘redoxome’ (Figure 2E). Here, high-sensitivity mass spectrometry is combined with differential thiol-trapping and isotope-coded affinity tagging (ICAT). Redox proteomic methods enable the identification of: (i) target proteins that undergo oxidative modifications, (ii) the nature of these modifications, and (iii) the exact residue modified within proteins [9]. Furthermore, the dose-dependent labelling of the proteome with low and high levels of a thiol-reactive probe allows the quantification of hyper-reactive cysteines, enriched with low-dose labelling, along with less reactive cysteine sites that require higher concentrations for labelling [79]. However, the ability of redox proteomics to identify a given oxidation status of certain cysteines is limited by: (i) the overall lower abundance of cysteine residues, (ii) a requirement for the appropriate proteolytic digestion of proteins into cysteine-containing peptides suitable for detection by mass spectrometry, (iii) the diversity of possible redox modifications, and (iv) the labile and dynamic nature of these redox modifications.

Application of a redox proteomic approach (OxICAT) to flies during ageing surprisingly found no difference in bulk thiol redox state between young and old flies, with the majority of cysteines present at ∼10–15% oxidised [80]. However, the study detected a strong oxidising shift upon fasting, showing that the fly thiol redoxome is physiologically responsive to nutritional interventions, and that these changes are detectable by OxICAT [80]. The samples consisted of female head and thorax tissue, as the abdomens were excluded to avoid confounding effects from differences in egg production with age. Consequently, some important metabolic tissues associated by the regulation of lifespan (e.g. gut, fat body) were not covered in the analysis. Also, the approach was an unbiased tryptic digest, and only a fraction of possible cysteines from the whole fly ‘thiome’ were captured (<1%, based on an *in silico* digest of the whole fly proteome). Therefore, although no bulk changes were detected, the redox regulation of specific proteins playing a role in the ageing process is still entirely possible, and needs further investigations using more targeted approaches. Here, the impact of redox-sensitive cysteine residues on ageing can be investigated through site-directed mutagenesis, for instance the generation of knock-in mutant flies, where a specific cysteine is replaced with a redox-inert serine (Figure 2F).

## Summary

Our ageing populations are associated with a rise in age-related diseases, challenging our social, economic and healthcare systems. Therefore, we urgently need to improve our biological understanding of the mechanisms underlying ageing. Amongst the numerous hypotheses to explain the ageing process, a causal involvement of ROS has been proposed. Initially, ROS was only recognised as causing oxidative damage, leading to cellular dysfunction and implicated as a driver of ageing. However, genetic overexpression of enzymes involved in antioxidant defences or dietary supplementation with antioxidants did not generally lead to lifespan extension. The observation that ROS also fulfil second messenger-like functions by modifying redox-sensitive cysteines in proteins changed the initial idea that the ROS-mediated accumulation of damaged macromolecules was responsible for ageing. Current evidence suggests that a pro-oxidising shift in cellular redox state may contribute to ageing.

Models such as *Drosophila* are valuable to study the ageing process, not only because they exhibit a comparatively short lifespan, but also because they can be easily genetically manipulated, which opens a window to study the role of ROS/cellular redox status and ageing. For instance, the advent of CRISPR enables a more widespread generation of targeted knock-in mutants for selected redox-active cysteines. Several of these redox-sensitive cysteines could so far be identified in mammalian systems, mainly by using cell culture models, but the existence of these respective redox switches under physiological conditions is still lacking and can be further investigated by combining the *in vivo Drosophila* model with advanced modern techniques available in the redox field.

## Perspectives

- Importance: We are living in an ageing society associated with an increase in age-related diseases, therefore we need better mechanistic insight into the ageing process itself.
- Current understanding and challenges: The ageing process is complex and still poorly understood. Considerable evidence exists that site-specific ROS generation and redox signalling processes are involved in regulating survival.
- Future directions: The rational design and combination of techniques to allow: (i) the measurement of specific ROS in a site-directed, compartment-, and tissue-specific manner *in vivo* in models such as *Drosophila*, (ii) the identification and manipulation of redox-sensitive cysteines *in vivo*, and (iii) further biochemical insight into the exact nature of the redox modifications, will lead to a better mechanistic understanding of the involvement of redox signalling events in the ageing process.

## Open Access

Open access for this article was enabled by the participation of Imperial College London in an all-inclusive *Read & Publish* pilot with Portland Press and the Biochemical Society under a transformative agreement with JISC.

## Abbreviations

ANT: adenine nucleotide translocase
AOX: alternative oxidase
CoQ: coenzyme Q
DCFDA: 2′,7′-dichlorofluorescin diacetate
ETC: electron transport chain
G6PDH: glucose-6-phosphate dehydrogenase
GCL: glutamate-cysteine-ligase
GR: glutathione reductase
GSH: glutathione (reduced)
GSSG: glutathione disulfide (oxidised)
H_2_O_2_: hydrogen peroxide
ICAT: isotope-coded affinity tagging
mtDNA: mitochondrial DNA
NAC: *N*-acetyl-cysteine
NDI1: internal NADH dehydrogenase
NOX: NADPH oxidase
O_2_^•−^: superoxide
PPP: pentose phosphate pathway
Prx: peroxiredoxin
RET: reverse electron transport
ROS: reactive oxygen species
SOD: superoxide dismutase
Trx: thioredoxin
TrxR: thioredoxin reductase
